# Do benzodiazepines reduce the efficacy of transcranial magnetic stimulation?

**DOI:** 10.1177/10398562241229623

**Published:** 2024-02-01

**Authors:** Lana Tran, Lisa Hahn, Shane Gill, Felicity Ng, Patrick Clarke, Tom Paterson, Cherrie Galletly

**Affiliations:** Psychiatry Trainee, School of Medicine, Discipline of Psychiatry, 94695 University of Adelaide, Adelaide, SA, Australia; Research Officer, Ramsay Clinic Adelaide, 1051 Ramsay Health Care (SA) Mental Health Services, Adelaide, SA, Australia; Consultant Psychiatrist, Ramsay Clinic Adelaide, 429218 Ramsay Health Care (SA) Mental Health Services, Adelaide, SA, Australia; Director of Psychiatry Training, Adelaide, SA, Australia; and School of Medicine, Discipline of Psychiatry, University of Adelaide, Adelaide, SA, Australia; Consultant Psychiatrist, School of Medicine, Discipline of Psychiatry, University of Adelaide; and Ramsay Clinic Adelaide, Ramsay Health Care (SA) Mental Health Services, Adelaide, SA, Australia; Emerita Professor, School of Medicine, Discipline of Psychiatry, University of Adelaide, Adelaide, SA, Australia; and Consultant Psychiatrist, Northern Adelaide Local Health Network, Adelaide, SA, Australia

**Keywords:** Major depressive disorder, treatment resistant, transcranial magnetic stimulation, benzodiazepine

## Abstract

**Objective:**

To investigate the effect of concomitant use of benzodiazepines on the efficacy of repetitive transcranial magnetic stimulation (rTMS) in patients with treatment-resistant major depressive disorder (TR-MDD).

**Methods:**

This is a retrospective study comparing rTMS treatment outcomes between patients taking benzodiazepines (*n* = 59) and those who were not (*n* = 136). Participants completed the HAM-A, HAM-D17, MADRS and ZUNG at baseline and at the end of treatment.

**Results:**

Patients taking benzodiazepines during rTMS treatment did not show any difference in partial response, response or remission rates compared to patients not treated with benzodiazepines. There was a significant decrease (*p* < .0001) in depression and anxiety scores from baseline to post-treatment among both groups.

**Conclusions:**

Concomitant benzodiazepine treatment had no effect on the efficacy of rTMS treatment of TRD, contrary to previous research.

Major depressive disorder (MDD) is a leading cause of disability and functional impairment.^1^ Treatment-resistant depression (TRD) is defined as inadequate response to at least two adequate antidepressant trials.^2^ The STAR*D study^3^ evaluated sequential treatments for MDD and found diminishing remission rates with each step of treatment, along with increasing side effects and treatment dropout.^4^

Comorbidity with anxiety disorders is common in MDD. A recent study found that 69% of 248 patients with TRD treated with rTMS had at least one comorbid anxiety disorder.^5^ Patients with MDD and comorbid anxiety have significantly more disease burden, greater side effect burden and poorer outcomes than those without comorbid anxiety.^6^ Repetitive transcranial magnetic stimulation (rTMS) is an effective, non-invasive, treatment for MDD.^7^ A study has also found rTMS to be effective for generalised anxiety disorder,^8^ although did not examine for the potential impact of medications.

Benzodiazepines have anxiolytic, anticonvulsant, muscle relaxant and sedative actions.^9^ In 2021, around 5.2 million benzodiazepine scripts were dispensed to 1.4 million patients in Australia.^10^ Despite the risk of dependence, benzodiazepines have evidence of benefit in the management of anxiety disorders.^11^ In patients with comorbid anxiety and MDD, they can be prescribed for symptoms associated with both conditions.

However, previous studies have suggested that concomitant benzodiazepine use may reduce the efficacy of rTMS treatment.^12–14^ Kaster et al.^12^ investigated patterns of rTMS treatment response (*n* = 388) comparing 10 Hz rTMS stimulation with intermittent theta burst stimulation (iTBS) and found that benzodiazepine use was associated with a lower likelihood of being in the ‘rapid response’ group. A retrospective chart review^13^ explored the outcomes with rTMS in 181 patients (72 were taking a benzodiazepine) and reported that benzodiazepines were associated with lower self-reported improvement in MDD symptoms. Deppe et al.^14^ retrospectively investigated rTMS outcomes among patients treated with lorazepam and found a significantly lower response rate to rTMS (18%) compared with the non-lorazepam group (38%). No association with frequency or dosage of lorazepam was found. By contrast, a study by Fitzgerald et al.^15^ found no difference in response rates between patients taking benzodiazepines or not (benzodiazepine users: 25% vs non-users: 24.8%).

There is also a possibility that medications may have a synergistic effect on rTMS outcomes. Hebel et al.^16^ found that there was no negative impact of lithium, lamotrigine and valproic acid, and there were better outcomes for lithium, lamotrigine and the combination of lithium and lamotrigine in response and remission rates. Resting motor threshold was significantly higher for patients taking valproic acid. A study by Hunter^13^ found no difference in TMS response rates or symptom improvement in 58 patients receiving non-benzodiazepine anti-epileptics compared to 123 patients who were not taking these medications.

Given the lack of consistency in previous research, the aim of this study was to further investigate the efficacy of rTMS for patients with TRD who are using benzodiazepines.

## Methods

This retrospective study used pooled data from two separate studies previously published by the same research group.^18,19^ These studies investigated the efficacy of different TMS protocols delivered to the dorsolateral prefrontal cortex (DLPFC) either on both sides (bilateral; BL) or on the right side (unilateral; UL) (Table 1).

**Table 1.** Treatment protocols

Study 1^18^	
Group 1 (*n* = 49)	Group 2 (*n* = 42)
BL treatment Duration 15 min each side Three times per week for 6 weeks (18 treatments) No significant differences in depression scores between people receiving rTMS 3 days/week for 6 weeks; and 5 days/week for 4 weeks	BL treatment Duration 15 min each side Five times per week for 4 weeks (20 treatments)

Study 2^19^	
Group 1 (*n* = 68)	Group 2 (*n* = 36)
RUL treatment Duration 15 min Three times per week for 6 weeks (18 treatments) No significant difference in response or remission rates between people receiving right UL rTMS and bilateral rTMS for the treatment of depression	BL treatment Duration 15 min each side Three times per week for 6 weeks (18 treatments)

rTMS was administered using a MagPro R30 stimulator and MCF B65 figure 8 coils. The resting motor threshold was measured using standard visual methods.^17^ A template was prepared for each participant to ensure the coils were positioned correctly at each treatment session. In all studies, rTMS was delivered at 110% of the resting motor threshold (RMT). For high frequency treatment, rTMS (10 Hz) was delivered in 5-s trains with a 25-s intertrain interval to the left DLPFC. Low frequency treatment was applied as continuous low frequency (1 Hz) rTMS pulses. All four groups had similar improvement in anxiety and depression symptoms at treatment completion.^18,19^

These studies were uniform in terms of recruitment procedure, referral processes, patient population, psychiatric and eligibility assessments, and the administration of validated instruments. Patients were recruited and referred by private psychiatrists affiliated with a private mental health hospital in a metropolitan region in South Australia.

The diagnosis of a major depressive episode was confirmed using DSM-IV-TR criteria and the Mini International Neuropsychiatric Interview^20^ by trained research staff. Patients received rTMS in an outpatient setting and remained under the care of their treating psychiatrists during rTMS treatment. Treating psychiatrists were requested not to make any changes to medication immediately before or during the patient’s rTMS treatment. Patients were assessed by an rTMS psychiatrist who confirmed the patient’s suitability and undertook the mapping procedure.

Inclusion criteria included a diagnosis of a major depressive episode (DSM-IV-TR), English language proficiency, no prior rTMS treatment and private health insurance. All patients met criteria for TRD, that is, had failed treatment with at least two antidepressants at adequate dose and duration. Exclusion criteria included insufficient English comprehension for assessment, cranial metal plates, electromagnetic incompatible devices or implants, epilepsy, or having drug or alcohol withdrawal. Patients with comorbid psychiatric disorder or other medical comorbidities were not excluded.

Written informed consent was obtained from all participants. Rating scales were administered by a trained research psychologist. Of the 195 subjects, 59 patients were concurrently taking benzodiazepines during rTMS treatment, and 136 patients were not.

All patients were assessed immediately prior to receiving their first rTMS treatment and immediately after receiving their last treatment of their full course. Participants completed the Hamilton Anxiety Rating Scale (HAM-A),^21^ 17-item Hamilton Depression Rating Scale (HAM-D17),^22^ Montgomery–Åsberg Depression Rating Scale (MADRS)^23^ and ZUNG Self-rating Depression Scale (ZUNG).^24^ Treatment outcomes were categorised as response, partial response and no response, based upon STAR*D criteria. Response was defined as at least a 50% reduction in HAM-D score and partial response as 25%–50% reduction.^2,25^ Remission (defined as a HAM-D score of 7 or less) was included in the ‘response’ group.

Statistical analyses were performed with SPSS version 24; IMB Corp., 2016. Baseline differences between people who were untreated and treated with benzodiazepines were tested using either Pearson chi square tests (*x*^2^) or independent samples T test (*t*).

Differences in partial response and response rates between the ‘Benzodiazepine’ and ‘No-Benzodiazepine’ groups at the end of treatment were tested using Pearson chi square tests (*x*^2^).

A paired sample test was performed to assess any significant changes in mean scores on the depression and anxiety rating scales from baseline to week 6.

Comparison of changes in HAM-D, HAM-A, MADRS and Zung scores from baseline to week 6 for the ‘Benzodiazepine’ group and the ‘No Benzodiazepine’ group was undertaken using a two-way mixed model ANOVA. The F-tests were used to test for differences in clinical scale scores between rTMS groups and for interactions between rTMS groups over repeated measures. The current study did not perform stratified randomised matching for age, gender, and depression or anxiety severity at baseline; therefore, these factors were controlled for in the ANOVA test. Psychiatric comorbidities were not controlled for in the analyses; past research using the same study cohort showed that having a comorbid anxiety disorder did not affect rTMS treatment outcomes.^5^ Medical comorbidities such as high blood pressure and high cholesterol were not collected in the current study. Power analyses (two-tailed, post hoc) were performed for the chi square and ANOVA test using G*Power version 3.1.9.4.^26^

## Results

Table 2 reports descriptive statistics. There were significantly more women, and more patients with GAD, taking benzodiazepines. There was no association between benzodiazepine status and age, number of years depressed, current duration of depressive episode, episodic depression, number of previous antidepressants trials or previous ECT.

**Table 2.** Descriptive characteristics of people taking benzodiazepines and not taking benzodiazepines during rTMS treatment

	Not taking benzodiazepines (*n* = 136)	Taking benzodiazepines (*n* = 59)	Test*, p* value
Age (years) M (SD)^a^	48.16 (14.51)	52.19 (12.27)	*t* (193) = −1.86, *p* = .064
Gender			
Male	62 (45.6%)	16 (27.1%)	*x*^2^(1) = 5.10, *p* = .024
Female	74 (54.4%)	43 (72.9%)	
Total number of years depressed, M (SD)^a^	19.09 (14.12)	22.45 (11.30)	*t* (190) = −1.60, *p* = .112
Duration of current depressive episode (months), M (SD)^a^	25.23 (46.22)	13.71 (12.95)	*t* (39) = 1.82, *p* = .072
Episodic depression			
Yes	70 (51.5%)	32/58 (55.2%)	*x*^2^(2) = 5.39, *p* = .067
No (continuous)	66 (48.5%)	24/58 (41.4%)	
Antidepressant trials			
Less than five	32/131 (24.4%)	10/56 (17.9%)	*x*^2^(1) = 0.63, *p* = .427
More than five	99/131 (75.6%)	46/56 (82.1%)	
Previous ECT	59/135 (43.7%)	34/58 (58.6%)	*x*^2^(1) = 3.04, *p* = .081
Unipolar depression	109 (80.1%)	44 (74.6%)	*x*^2^(1) = 0.46, *p* = .497
Generalised anxiety disorder	45 (33.1%)	29 (49.2%)	*x*^2^(1) = 3.85, *p* = .050
Obsessive compulsive disorder	20 (17.7%)	3 (5.1%)	*x*^2^(1) = 2.80, *p* = .095
Post-traumatic stress disorder	15 (11.0%)	4 (6.8%)	*x*^2^(1) = 0.43, *p* = .512
Panic disorder (with agoraphobia)	13 (9.6%)	12 (20.3%)	*x*^2^(1) = 3.37, *p* = .066

^a^M = mean; SD = standard deviation.

There was no significant difference in response outcomes (χ^2^ (3) = 45.75, *p* = .191) among people who were treated with benzodiazepines compared with people who were not (Table 3).

**Table 3.** Response outcomes among people not taking benzodiazepines and people taking benzodiazepines after rTMS treatment

	Not taking benzodiazepines (*n* = 136)	Taking benzodiazepines (*n* = 59)	*x*^2^ value (df)	*p* value
Response outcome				
No response	52 (38.2%)	22 (37.3%)	4.75 (3)	0.191
Partial response	31 (22.8%)	14 (23.7%)		
Response	14 (10.3%)	12 (20.3%)		
Remission only	39 (28.7%)	11 (18.6%)	1.68 (1)	0.195

Partial response is at least 25% reduction in HAM-D score. Response is as at least 50% reduction in HAM-D score; HAM-D score ≤7 indicates remission; *n* = 195 provides 80% power (α = 0.05) to detect a medium effect *(η*^*2*^_*p*_ *=* 0.05) in response outcomes.

Table 4 shows a significant decrease (*p* < .0001) in scores on the depression and anxiety rating scales from baseline to post-treatment for both groups who were and were not taking benzodiazepines.

**Table 4.** Change in mean depression and anxiety scores between baseline and the end of treatment in people not taking benzodiazepines (*n* = 136) and taking benzodiazepines (*n* = 59)

	Baseline, M (SD)	Post (week 6), M (SD)	Mean change	*p* value
HAM-D 17-item				
Not taking BZD	20.01 (6.11)	12.11 (7.25)	7.9	<0.0001
Taking BZD	21.76 (5.57)	14.19 (8.12)	7.57	<0.0001
HAM-A				
Not taking BZD	19.01 (7.52)	12.79 (7.68)	6.21	<0.0001
Taking BZD	21.37 (6.59)	14.68 (8.37)	6.69	<0.0001
MADRS				
Not taking BZD	30.01 (7.84)	20.32 (11.55)	9.69	<0.0001
Taking BZD	31.93 (7.63)	22.75 (12.53)	9.18	<0.0001
Zung				
Not taking BZD	57.14 (7.01)	47.90 (10.93)	9.24	<0.0001
Taking BZD	57.64 (7.94)	49.87 (10.81)	7.77	<0.0001

HAM-D: Hamilton Depression Rating Scale; HAMA: Hamilton Anxiety Rating Scale; MADRS: Montgomery–Åsberg Depression Rating Scale; Zung: Zung Self-Rating Depression Scale.

There was no significant difference between the two comparison groups in depression and anxiety reduction; but there was a significant effect for time. There was no significant interaction between the treatment group and time. After adjusting for age, gender and baseline scores on the HAM-D, MADRS, HAM-A and Zung, no significant effect was found by the treatment group (Table 5).

**Table 5.** Change over time, by treatment group and interaction effects using ANOVA

Rating scale	Effect over time	Effect by treatment group	Time*treatment group
HAM-D 17-item	F (1, 193) = 186.98, *p* < .001	F (1, 193) = 0.69, *p* = .409	F (1, 193) = 0.08, *p* = .778
HAM-A	F (1, 193) = 105.57, *p* < .001	F (1, 193) = 0.97, *p* = .326	F (1, 193) = 0.14, *p =* .711
MADRS	F (1, 193) = 125.72, *p* < .001	F (1, 193) = 1.60, *p* = .208	F (1, 193) = 0.10, *p* = .761
Zung	F (1, 193) = 115.44, *p* < .001	F (1, 193) = 1.51, *p* = .220	F (1,193) = 0.88, *p =* .352

*n* = 195 provides 80% power (α = 0.05) to detect a medium effect *(η*^*2*^_*p*_ *=* 0.05) in scores on the depression and anxiety rating scales.

## Discussion

Consistent with Fitzgerald et al.,^16^ this study found that concomitant benzodiazepine use does not reduce the effectiveness of rTMS in patients with TRD. Similar to this study, Fitzgerald et al.^16^ categorised benzodiazepine use as a binary variable (i.e. a ‘yes’ or ‘no’) with no distinction made regarding dosage and frequency of use. A limitation of this approach is that it cannot detect effects related to dose or frequency of use, in contrast to previous work that explored a dose effect.^14^

Contrary to our findings, earlier studies^12–14^ found a negative impact of concomitant benzodiazepines. Hunter et al.^13^ theorised that the effect of benzodiazepines on rTMS efficacy may be related to stimulation site and frequency. In their study, all patients were given 10 Hz treatment to the left DLPFC, which was then changed to low frequency right-sided stimulation after 2 weeks if patients were unable to tolerate left-sided stimulation or showed inadequate response. The study found that after 2 weeks’ treatment, patients taking benzodiazepines showed poorer response. This could suggest that benzodiazepine use is correlated with poorer early response to rTMS, but this needs to be further explored.

Other important factors that may account for differences in results include the number and type of comorbidities, frequency and dosage of benzodiazepine, other medications, and substance use. Studies^5,27^ have found that comorbid anxiety disorders do not appear to impact outcomes when rTMS is used to treat TRD, but there may be factors associated with benzodiazepine use other than the nature of patients’ psychiatric disorders.

Since there were no sham treatment arms in our studies, the authors considered the possibility that patients in both groups may have spontaneously improved, rather than improvement being due to TMS. To investigate this, we compared our results with sham TMS outcomes in TMS trials that included a sham arm. O’Reardon et al.^28^ reported a sham response rate of 11% and sham remission rate of 11%. George et al.^29^ reported a sham response rate of 5% and remission of 4%. Our response rates (39% in both groups) and remission rates (29% and 19%) were considerably higher, indicating that these outcomes were unlikely to be due to spontaneous improvement unrelated to TMS.

Given that many patients with TR-MDD may be concomitantly using benzodiazepines, our data is important in evaluating inclusion criteria for patients who may benefit from rTMS and avoid unnecessary treatment delay to cease benzodiazepines and their withdrawal syndromes.

## Limitations

The dosage, frequency of use and type of benzodiazepine were not reported. This study was retrospective in nature and drew its cohort from two studies exploring different rTMS protocols. Due to this study being conducted in a private hospital outpatient clinic, results may be impacted by selection bias involving possible differences in the clinical characteristics of this demographic. Other variables potentially associated with benzodiazepine use such as alcohol use disorder were not explored.

## Conclusions

This study found no difference in the response of patients with TRD to rTMS between patients taking/not taking benzodiazepines concurrently with treatment. The response rates were consistent with those noted in other studies observing the efficacy of TMS in patients with major depression. Our findings suggest that concomitant benzodiazepine treatment should not be a contraindication to rTMS, and that these patients are likely to have similar outcomes as those not taking benzodiazepines. Further studies with larger datasets and more nuanced investigation of the impact of benzodiazepines and other medications are needed.
